# Accessibility and availability of smartphone apps for schizophrenia

**DOI:** 10.1038/s41537-022-00313-0

**Published:** 2022-11-16

**Authors:** Sam Kwon, Joseph Firth, Devayani Joshi, John Torous

**Affiliations:** 1 Department of Psychiatry, Beth Israel Deaconess Medical Center, Boston, MA USA; 2 Division of Psychology and Mental Health, University of Manchester, Manchester Academic Health Science Centre, Manchester, UK; 3 Greater Manchester Mental Health NHS Foundation Trust, Manchester Academic Health Science Centre, Manchester, UK

**Keywords:** Diseases, Biomarkers

## Abstract

App-based interventions have the potential to enhance access to and quality of care for patients with schizophrenia. However, less is known about the current state of schizophrenia apps in research and how those translate to publicly available apps. This study, therefore, aimed to review schizophrenia apps offered on marketplaces and research literature with a focus on accessibility and availability. A search of recent reviews, gray literature, PubMed, and Google Scholar was conducted in August 2022. A search of the U.S. Apple App Store and Google Play App Store was conducted in July 2022. All eligible studies and apps were systematically screened/reviewed. The academic research search produced 264 results; 60 eligible studies were identified. 51.7% of research apps were built on psychosis-specific platforms and 48.3% of research apps were built on non-specific platforms. 83.3% of research apps offered monitoring functionalities. Only nine apps, two designed on psychosis-specific platforms and seven on non-specific platforms were easily accessible. The search of app marketplaces uncovered 537 apps; only six eligible marketplace apps were identified. 83.3% of marketplace apps only offered psychoeducation. All marketplace apps lacked frequent updates with the average time since last update 1121 days. There are few clinically relevant apps accessible to patients on the commercial marketplaces. While research efforts are expanding, many research apps are unavailable today. Better translation of apps from research to the marketplace and a focus on sustainable interventions are important targets for the field.

## Introduction

Increasing access to care for people with schizophrenia remains a global health priority^1^. Given their scalable potential, digital mental health and especially smartphone-based solutions have become a topic of great interest^2^. In the last decade, research on smartphone apps for people with schizophrenia has expanded, with results suggesting uses cases around early diagnosis, real-time monitoring, psychoeducation, relapse prevention, and even therapeutic interventions^3–5^. But to what degree has this research translated into digital tools that patients today can access and utilize? This paper thus aims to review both the research and commercial marketplaces for apps for people with schizophrenia and explore their availability and accessibility.

Smartphone apps represent an important solution to increasing access to care for people with schizophrenia. A preponderance of data suggests that not only do people with schizophrenia own smartphones at high rates but that they also are interested in using them as part of their recovery^5–10^. Despite often raised questions by the public regarding whether smartphone-based monitoring or interventions may make people with schizophrenia more paranoid, there remains scant data to support this unfounded claim^11^. Rather, people with schizophrenia have themselves been at the forefront of developing and researching new advances in the uses of smartphones for their condition^12,13^.

The driving force behind the interest in smartphones centers on increased access to care. The simple reality of the lack of a mental health workforce able to deliver care to people with serious mental illness was clear before COVID-19, and now is even more apparent^14^. Given the enormous mental health gap in services, impacting all countries in the world, digital solutions that are scalable represent a critical target for increasing access to care^15^. As the unmet need for care for people with schizophrenia is at the higher end of the spectrum, the concomitant interest in smartphone apps for this condition is clear. Apps have been proposed as tools to help with screening and monitoring of schizophrenia as well as tools to offer on-demand as well as just-in-time adaptive interventions^16,17^; and an active area of research for the last ten years.

Beyond access, interest in these apps is also fueled by the potential of smartphone apps to deliver more holistic and eclectic treatments beyond those readily accessible today. For example, smartphone apps can facilitate cognitive remediation treatments, peer support, cognitive behavioral therapy for psychosis, and other services that may be challenging for patients to find today even in well-resourced countries^5^.

However, despite this potential, it is also clear from evidence across all of digital health that smartphone apps alone are not a panacea. Concerns around privacy, effectiveness, engagement, and clinical integration are now well-recognized barriers for all health-related apps, including mental health apps^18^. Even in 2022, mental health app privacy concerns continue to make national news^19^ and there is rising awareness for high quality studies that assess the impact of these apps against appropriate control conditions^20,21^. While practical experience suggests that apps for people with schizophrenia have not yet transformed care in 2022 and that there is no well-defined or practical distinction between clinician-prescribed apps and self-prescribed apps related to schizophrenia, little is actually known about the current state of the field or the availability of apps for practical patient use.

To understand the current state of apps for schizophrenia, this review aims to catalog these apps developed in the research space based on platforms and assess their current availability. In parallel, this review also aims to investigate the quality of apps currently offered on the Apple and Android marketplaces and assess any overlap or differences in features between the research apps. Towards understanding factors that may impact availability, this review focuses on apps as platforms rather than the clinical results of any one study.

## Methods

### Research articles search and a literature review

To analyze the current research on smartphone apps for individuals with psychosis, we searched recent reviews^22,23^, the gray literature, and standard academic databases including PubMed and Google Scholar. This intentionally surface-level search strategy of the academic literature was intentionally employed as a primary purpose of this dual review (which also searched app stores, see below) was to assess and compare the apps used in the most easily-identified research studies, to those available on publicly-accessible app marketplaces.

A search of PubMed and Google Scholar was performed on August 17, 2022, using the following search algorithm: (“smartphone*“ or “mobile phone*“ or “cell phone”)) AND (“app” or “apps” or “application” or “applications”)) AND (“schizophrenia” or “schizo” or “psychosis” or “psychotic”). However, a primary purpose of the review was to gain an assessment of the extent to which apps in readily-accessed research studies are aligned with those apps widely available on the marketplace, and there is no reason to believe that any missed studies more difficult to identify would significantly impact our data collection.

A total of 309 articles were revealed. After removing duplicates, a total of 264 articles remained.

Two authors (SK and DJ) screened each title/abstract for eligibility using the Covidence systematic review management tool^24^. Articles were excluded if they (1) delivered an intervention that is not mobile app-based (2) were unrelated to psychosis.

**Fig. 1:**
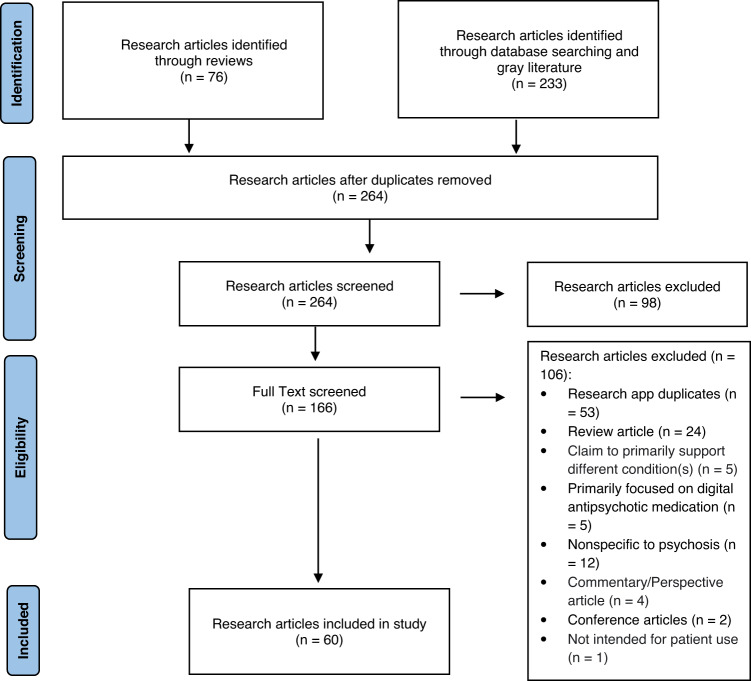
Flow diagram of research articles screening.

A total of 166 articles were reviewed in full text by 3 authors (SK, DJ, and JT) using the following inclusion/exclusion criteria: articles were excluded if they (1) utilized the same research app as other articles (2) were review articles (3) were non-specific to psychosis (4) were commentary or perspective articles (5) were conference abstracts (6) used the apps not intended for patients’ use, (7) were primarily focused on supporting different condition(s) such as smoking, or (8) were primarily focused on digital antipsychotic medication with the smartphone app only supporting the digital medicine. Consequently, a total of 60 research apps were included (Fig. 1). In this article, the term “research apps” will be used solely when referring to the mobile apps utilized by the academic article.

### Public app marketplace mobile application search and an app audit

To analyze the current marketplace for psychosis apps, a search of the Apple App Store and Google Play App Store was conducted on July 24, 2022. There were no inclusion criteria for the commercial mobile application search since the aim of this study was to determine the most accessible and easily obtainable psychosis apps for a layperson. The terms “Schizophrenia” and “Psychosis” were entered. A total of 675 apps returned, which was reduced to 537 apps after duplicates across Apple and Android were eliminated. In the first phase of screening, returned apps were excluded if they (1) were not available in English (2) developed to include contents unrelated to mental health (eg dating apps) (3) were non-specific to schizophrenia or psychosis (4) claimed to primarily support different condition(s) (5) intended for test preparation (6) intended for non-patient use, and (7) cost more than $10.00 to download; this price limit was set on the premise that an app should be economically accessible and determined based on our team’s clinical experience and patient interactions.

Subsequently, a total of 512 apps were excluded. Two authors (SK and JT) downloaded and used the remaining 25 apps and reviewed each app for approximately 30 min. These apps were then further screened and excluded if they (1) contained outdated information as determined by a patient advisory panel (2) were non-functional (3) required access code, and (4) contained potentially dangerous or stigmatizing information as determined by a patient advisory panel. Outdated information included references and diagnosis excluded from the DSM-5. Potentially dangerous or stigmatizing information included home remedy recommendations without a proper side effects warning and a phrase that perpetuates negative labeling and perception. Subsequently, a total of six marketplace apps were included. The term “marketplace apps” will be utilized exclusively when referring to the psychosis mobile apps returned on the public app marketplace.

### Therapeutic, monitoring, and psychoeducation (TMP) classification

There is no established nosology for the categorization of the main features or functionalities of research apps and marketplace apps. Thus, we opted to categorize these features or functionalities broadly into three categories: therapeutic, monitoring, and psychoeducation. For ‘therapeutic’ we counted any features that aim to improve symptoms, behavior or cognitive functioning, such as psychotherapy, skills training, peer support, and tailored daily activities. For “monitoring” we counted any features that help patients to track symptoms, treatment progress, or medications. For ‘psychoeducation’, we counted any features that offered reference or didactic information. Using this nosology, it is possible for the same app to offer therapeutic, monitoring, and psychoeducation functionalities.

### Specific platform and non-specific platform

We further classified apps as built or running on specific vs non-specific platforms. A specific platform would be a custom app designed to run only that program. For example, an app built on specific platform would only provide psychoeducation regarding schizophrenia. A non-specific app may be a broad survey platform customized with relevant clinical content or an app platform customized to support specific content. For example, a non-specific app could be a survey-administering app that brings up different question sets based on a set-specific code. A researcher may create a set of questions relevant to schizophrenia patients and administer those questions using a non-specific app.

### Data analysis process

A total of 60 research articles and their respective research apps were analyzed, and the data extracted including availability on the public app marketplace, access code requirement, supporting study authors and year, research app example features, TMP framework categorization, and specific vs non-specific platform. All research apps available for download were assessed to evaluate accessibility and categorized as restricted access (requires access code) or full access (does not require an access code).

A total of six marketplace apps were analyzed. Data analyzed included the date of last update, app descriptions, and the TMP framework categorization. The six marketplace apps were also rated based on the M-health Index and Navigation Database (MIND) derived from the American Psychiatric Association’s app evaluation framework^25,26^. MIND is the largest public database that allows any users to make an informed decision in choosing a mental health app. The rating included 105 objective questions pertaining to app origin, functionality, and accessibility; privacy and security; evidence and clinical foundations; features and engagement; interoperability and data sharing^27^.

### Statistical analysis

The download availabilities of specific and non-specific research apps were compared using the chi-square test. Statistical significance was defined as *P* < 0.05.

## Results

**Fig. 2:**
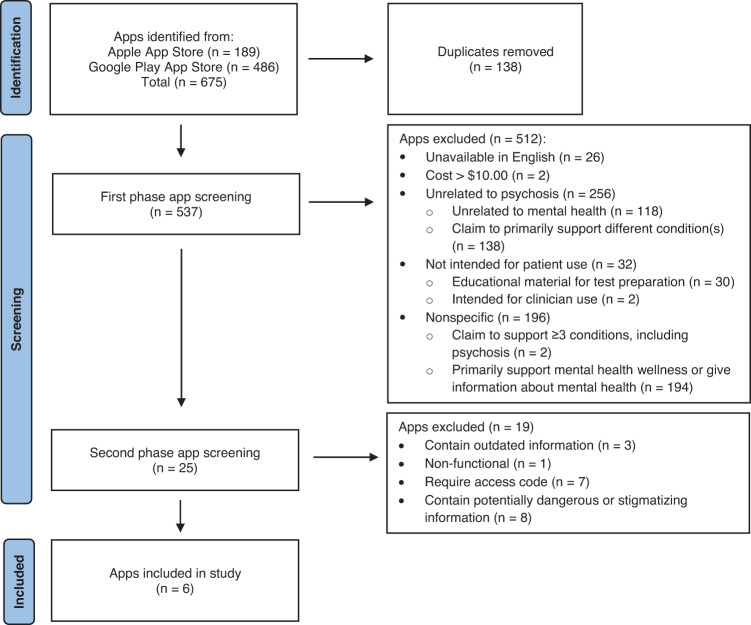
Flow diagram of marketplace apps screening.

A total of 537 unique marketplace apps were identified on the United States Apple App Store and Google Play App Store when the terms “Schizophrenia” and “Psychosis” were searched. As shown in Fig. 2, 256 of 537 apps (47.7%) were not related to psychosis, and 32 of 537 apps (6.0%) were not developed to be used by individuals with psychosis. There were two apps (0.4%) that specifically stated they supported psychosis in addition to two or more different conditions, including eating disorder, PTSD, generalized anxiety disorder, plus 27 other conditions, and 194 apps (36.1%) that claimed to support mental health wellness or provide information about mental health. Only 25 apps (4.7%) claimed to primarily support psychosis. However, of the 25 apps, one app was non-functional, seven were inaccessible without an access code, three apps contained outdated information such as reference to non-current diagnosis like Disorganized, Catatonic and Undifferentiated schizophrenia which have been removed from the DSM-5 for over five years. Eight apps contained stigmatizing or dangerous information such as telling users to “remember that it’s [schizophrenia] all in your head” or providing herbal supplement advice without discussing potentially dangerous medication interactions. Outdated information included references and diagnoses that had been excluded from the DSM-5, and dangerous information included a list of medications that can cause harmful drug interactions with antipsychotics. The accessibility to obtaining an access code was limited as users had to either (1) contact the app developer (2) contact the research group (3) partake in the research study or (4) be a patient at a specific clinic. Thus, a total of six easily accessible, appropriate, and psychosis-specific marketplace apps (1.1%) were evaluated using the MIND framework (Fig. 2).

### Marketplace App

Of the six easily accessible, appropriate, and psychosis-specific marketplace apps, five apps solely presented psychoeducation (83.3%). Only one app, which was last updated 1587 days ago (4.34 years ago), offered therapeutic and monitoring functionalities without providing psychoeducation. All six apps were not updated frequently with the average time since last updated being 1121 days (3.07 years ago)—well exceeding the 180 days update metric adopted by the American Psychiatric Association, which prompts a concern for an app quality and safety^28^ (Table 1).

**Table 1:** Accessible, appropriate, psychosis-specific marketplace apps.

App Name	Download available in Apple App Store?	Download available on Google Play App Store?	T	M	P	Last Updated Date	App Description
PIER	No	Yes	−	−	+	April 8, 2019	The app provides information about psychosis symptoms, management, and intervention.
Schizophrenia HealthStorylines	Yes	Yes	+	+	−	March 20, 2018	The app offers a platform to record symptoms, mood, and medications and get connected to peer support.
Schizophrenia	No	Yes	−	−	+	October 28, 2017	The app provides 3D animations of information about schizophrenia causes, symptoms, diagnosis, and treatment options.
Schizophrenia Treatment- Remedies for Schizophrenia	No	Yes	−	−	+	March 24, 2019	The app offers information about natural remedies for schizophrenia.
Schizophrenia Disease	No	Yes	−	−	+	March 12, 2021	The app provides information about signs and symptoms, causes, treatment, diet, and risk factors for schizophrenia.
Coping with Schizophrenia	No	Yes	−	−	+	March 17, 2021	The app provides a link to peer support group and information about coping skills for schizophrenia.

*T* therapeutic, *M* monitoring, *P* psychoeducation.

All six of these apps were free to download with one offering in-app purchases (16.7%) and five totally free (83.3%). One of the six apps was available on the Apple App Store, and all six apps were available on the Google Play App Store. Of the six apps, four apps had a privacy policy and only one app allowed users to delete data. Moreover, only one app allowed users to opt-out of data collection, and none of the apps claimed to meet Health Insurance Portability and Accountability Act, 1996 (HIPPA) – established to protect an individual’s protected health information^29^. Only one app included a published supporting study examining the app’s feasibility^30^.

### Research App Feature

Of the 60 unique academic articles on smartphone apps for psychosis, 31 articles utilized apps designed with a psychosis-specific platform, and 29 articles used apps built on a non-specific platform to deliver psychosis-specific features. 50 of the 60 research apps provided symptom monitoring features while 30 research apps exclusively offered monitoring features, 14 research apps offered therapeutic and monitoring features, 3 research aps offered monitoring and psychoeducation features, and 3 research apps offered therapeutic, monitoring, and psychoeducation features, see Tables 2, 3. Although nine research apps included psychoeducation in addition to monitoring or therapeutic features, no research app solely provided psychoeducation (Tables 2, 3).

**Table 2:** Studies of psychosis research apps with psychosis-specific platform.

App Name	Can you download on public app stores?	Does the app require access code or special credentials?	T	M	P	Supporting Study (Author, year)	Example Features from One Paper on That App. Note: All Features may not be discussed or covered.
A4i*^48,49^	Yes	Yes	+	+	+	Kidd et al. 2021^48^	The A4i app aims to support by providing seven functionalities that include daily activities, passive data collection, peer support, and self-management. The goal of the study is to investigate the feasibility of A4i to supplement limited service.
Actissist±*^50–52^	Yes	N/A	+	+	+	Berry et al. 2020^50^	The Actissist app delivers cognitive behavioral therapy for early psychosis patients. Some changes in the app include non-directive content, recovery videos, calming exercises, psychoeducation, and goal-setting features.
CBT2go App*^53–55^	Yes	Yes	+	+	−	Granholm et al. 2020^53^	mCBTn is composed of weekly in-person groups, and the CBT2go app that tracks activities, aids homework assignment adherence, and prompts individualized activities. The study outcomes showcase decreased negative symptoms in the mCBTn group.
ClinTouch*^56–58^	No	N/A	+	+	−	Lewis et al. 2020^56^	The ClinTouch app provides active self-monitoring platform to detect signs of relapse and gives feedback to promote self-management. The study showed decrease in PANSS scale in ClinTouch group compared to TAU and that the app is feasible in detecting signs for relapse.
+Connect±*^59,60^	Yes	N/A	+	−	−	Lim et al. 2020^59^	The +Connect app aims to reduce loneliness in young adults with early psychosis by providing contents to exercise positive interpersonal skills. The results indicate that using the app decreased loneliness measured via the University of California Loneliness scale.
CrossCheck*^61–63^	No	N/A	−	+	−	Buck et al. 2021^61^	The CrossCheck app collects EMA surveys and passive accelerometers data. Study outcomes demonstrate that EMA identified mood and symptoms changes prior to the relapse.
ExpiWell^64^	Yes	Yes	+	−	−	Taylor et al. 2022^64^	The ExpiWell app delivers CBT-based modules to aid with sleep problems in patients with psychosis. The modules consist of six weekly sessions. The study outcomes indicate that the participants found the app to be acceptable and feasible.
ExPRESS*^65,66^	No	N/A	−	+	−	Eisner et al. 2019^65^	The ExPRESS app provides weekly assessments that collect psychotic signs and symptoms. The study showed that the app-based symptom assessments were practical and valid.
FOCUS*^67–69^	No	N/A	+	−	−	Achtyes et al. 2019^67^	The FOCUS app provides illness management guidance in 5 areas: sleep, mood, social, medication, and voices. The results indicate that a 50% of the app access occurred when the clinic was closed and that ‘high utilizers’ consist of a particular demographic subgroup.
GRASP^70^	No	N/A	+	−	−	Sedgwick et al. 2021^70^	The GRASP app is delivered with the group therapy sessions to provide weekly homework to improve cognitive functions. The results indicate that the app intervention with the therapy sessions were feasible and helpful.
Heal Your Mind (HYM)^71^	Yes	No	−	+	−	Kim et al. 2018^71^	HYM is a case management and symptom monitoring app for young patients with early psychosis. The study highlights that the majority of participants found the app easy to use and were content with the app.
iVoice*^72,73^	No	N/A	−	+	−	Smelror et al. 2019^72^	The iVoice app helps patients with early onset psychosis with auditory verbal hallucination management. The study results indicate that the real-time monitoring of auditory verbal hallucination can enhance diagnostic resolution.
MASS*^74–76^	No	N/A	+	+	−	Fulford et al. 2022^74^	The MASS app seeks to address social impairment by offering social skills training and administering EMA surveys. The study findings indicate that there is no association between cognitive functions and engagement or outcomes of the MASS intervention.
MedActive^77^	No	N/A	+	+	−	Kreyenbuhl et al. 2019^77^	The MedActive app aims to improve antipsychotic medication adherence for individuals with schizophrenia by providing tailored reminders and feedback. The study results indicate that the app was found to be feasible and usable by the patient participants.
MONEO*^31,32^	No	N/A	+	−	−	Krzystanek et al. 2020^31^	The MONEO app seeks to improve cognitive functions in schizophrenia by providing cognitive training exercises. The results indicate that the cognitive training offered by the app is feasible for schizophrenia patients.
My Journey 3*^78,79,80^	No	N/A	−	+	+	Steare et al. 2021^78^	The My Journey 3 app aims to support psychosis patients by providing self-management interventions. The features include relapse prevention plan, symptom and medication tracker, and psychoeducation. The study outcomes indicate that the participants found the app to be useful and helpful.
MCI-S^81^	No	N/A	+	−	+	Han et al. 2022^81^	The MCI-S app offers metacognitive intervention program and information about psychosis. The study outcomes show that the app in addition to the weekly mentoring program improved psychotic symptoms and social functioning compared to the group that solely used the app.
PEAR-004^21^	Yes	Yes	+	+	−	Ghaemi et al, 2022^21^	PEAR-004 is a self-management app that provides 10 categories skills, surveys, modules, and contents. The results demonstrate no difference in the total PANSS score between the PEAR-004 and sham control groups.
PLAN-e-PSY^82^	No	N/A	−	+	−	Haesebaert et al. 2021^82^	The PLAN-e-PSY app study will investigate whether the individualized goal setting and tracking app enhances functioning in FEP patients compared to TAU.
PRIME-EPI-MINN*^83,84^	Yes	Yes	+	+	−	Schlosser et al. 2018^83^	The PRIME app targets to enhance motivation and quality of life in patients with first-episode psychosis by providing peer-support, individualized daily activities, and coach connection. The study demonstrates that the PRIME intervention improved depression, pessimistic beliefs, motivations, and self-efficacy.
Psychotherapy app^85^	No	N/A	+	+	+	Barbeito et al. 2019^85^	The Psychotherapy app consists of five modules (psychoeducation, symptom recognition and relapse prevention, problem-solving, mindfulness, and contact wall) to improve accessibility for FEP patients. The study will determine whether the app intervention improves symptoms and quality of life compared to TAU.
ReMind^86^	No	N/A	−	+	−	Hui et al. 2021^86^	The ReMind app aims to predict relapse in schizophrenia patients by administering LNS and VPT and providing psychosocial assessments.
ReMindCare*^87,88^	Yes	Yes	−	+	−	Bonet et al, 2020^87^	The ReMindCare app provides daily questionnaires to evaluate patient status. The study results indicate that ReMindCare intervention group experienced fewer relapse and visits to urgent care compared to TAU.
Schizophrenia HealthStoryline^30^	Yes	No	+	+	−	Torous et al. 2017^30^	The Schizophrenia HealthStoryline app provides symptoms, mood and medications trackers and peer support. The study results found that the overall engagement with the app was low.
SlowMo/Native^89^	No	N/A	+	−	+	Hardy et al. 2018^89^	The SlowMo platform, a web app with 8 face-to-face sessions of psychoeducation and skills practice, is used alongside a native mobile app that is synchronized for daily use. The study findings emphasize that the user-centered designed app can complement the traditional therapy.
TechCare*^90–92^	No	N/A	+	+	−	Gire et al. 2021^90^	The TechCare app aims to reduce relapse by providing real-time therapy and symptom monitoring. The study outcomes showcase a potential of the app to increase accessibility in care and be feasible to support psychosis.
Temstem±^93^	Yes	N/A	+	−	−	Jongeneel et al. 2018^93^	The Temstem app offers language games with an aim to allow individuals with psychosis to have control over and suppress auditory verbal hallucinations.
Unnamed App^94^	No	N/A	−	+	−	Sturup et al. 2017^94^	Sturup et al, 2017 aims to conduct a TAILOR trial in which a tapering/discontinuation group of patients will utilize an app to monitor early signs of relapse. This study will determine the effect of tapering/discontinuing antipsychotic medications in early stages of schizophrenia.
Unnamed App^95^	No	N/A	+	+	−	Weintraub et al. 2022^95^	Weintraub et al, 2022 conducted a study for the use of mobile app in addition to the CBT group sessions for youth with psychosis or severe mood disorder. The app offers group session review, skills practice, and symptom monitoring. The results indicate that the participants found the app and the group sessions to be feasible and acceptable and the intervention improved psychotic symptoms and functioning.
weCOPE*^96,97^	No	N/A	+	+	−	de Almeida et al. 2018^96^	The weCOPE app offers four modules: symptom monitoring, problem-solving, anxiety-management, and goal setting. The results indicate that the weCOPE app has a potential to support schizophrenia patients.
WorkingWell*^98,99^	No	N/A	+	−	−	Nicholson et al. 2018^98^	The WorkingWell app aims to support individuals with serious mental illness to manage stress and anxiety occurring in workplaces by providing ways to manage interpersonal relationships and cope with challenging social situations.

*T* therapeutic, *M* monitoring, *P* psychoeducation, *mDOT* modified directly observed therapy, *mCBTn* mobile assisted cognitive behavioral therapy for negative symptoms, *TAU* treatment as usual, *MERIT* metacognitive reflection and insight therapy, *EMA* ecological momentary assessment, *ESM* experience sampling method, *LSN* letter-number span, *VPT* visual patterns test, *CHR* clinical high risk, *PANNS* positive and negative syndrome scale, *FROGS* functional remission of general schizophrenia scale, *CBT* cognitive behavioral therapy, ± download unavailable in the US, * articles with a maximum of two other representative articles.

**Table 3:** Studies of psychosis research apps with non-specific platform.

App Name	Can you download on public app stores?	Does the app require access code or special credentials?	T	M	P	Supporting Study (Author, year)	Example features from one paper on that app. Note: all features may not be discussed or covered.
AiCure^100^	Yes	Yes	−	+	−	Bain et al. 2017^100^	The AiCure app, which measures medication adherence, is compared to mDOT. Study outcomes indicate that the AiCure-intervention increased medication adherence compared to the mDOT-intervention.
Beiwe*^101–103^	Yes	Yes	−	+	−	Barnett et al. 2018^101^	The Beiwe app collects passive data, including GPS data, SMS text and call logs, to detect anomalies in patient behavior. The study findings showed that the app has the potential to predict relapse in schizophrenia patient.
BrainHQ(CLIMB)*^104–106^	Yes	No	+	+	−	Dabit et al. 2021^104^	The CLIMB program includes weekly group sessions, peer-to-peer instant messaging, social cognitive training, and EMA. The BrainHQ app was used with the goal to improve social cognition in schizophrenia patients. The results indicate that the CLIMB-intervention improved social functioning compared to active control.
CORE^107^	No	N/A	+	−	+	Ben-Zeev et al. 2021^107^	The CORE app, developed based on the GGtude platform, provides game-like exercises to promote adaptive beliefs and give psychoeducation. The results showed that the app is found to be feasible and effective in reducing psychotic symptoms while aiding in recovery for patients with serious mental illness.
DIALOG + *^108–110^	Yes	No	−	+	−	Fichtenbauer et al. 2019^108^	The DIALOG + app adds to the clinical consultation by providing psychometric scale and goal setting features for patients with psychosis. The study outcomes underscore that using the app reduced schizophrenia symptoms.
DiAPAson^111^	No	N/A	−	+	−	Zarbo et al. 2022^111^	The DiAPAson intervention includes the Actigraph device and the DiAPAson app that administers an ESM questionnaire. The study outcomes indicate that the participants with schizophrenia had reduced adherence to the intervention compared to the control group.
EAR±*^112,113^	Yes	N/A	−	+	−	Minor et al. 2022^112^	The EAR app records audio in patient’s daily life passively. The results indicate that Tailored MERIT with the EAR app decreased negative beliefs and disorganized symptoms compared to standard MERIT with the EAR app.
EasyM^114^	No	N/A	−	+	−	Moran et al. 2017^114^	The EasyM app administers an EMA questionnaire to assess negative symptoms in schizophrenia patients. The study findings showcase that the EMA questionnaire is a suitable method to assess negative symptoms of schizophrenia.
eB2^115^	No	N/A	−	+	−	Lopez-Morinigo et al. 2021^115^	The eB2 app is a passive EMA app that collects mobility, physical activity, sleep, social activity data. The study outcomes indicate that schizophrenia patients showed low acceptability of using the eB2 app.
EMPOWER*^116,117^	No	N/A	−	+	−	Gumley et al. 2022^116^	The EMPOWER intervention aims to recognize and manage relapse in schizophrenia using the self-management mobile app and peer support. The study outcomes demonstrate that the EMPOWER group had fewer relapse and reduced fear of relapse compared to TAU.
Ginger.io*^118,119^	Yes	Yes	−	+	−	Niendam et al. 2018^118^	The Ginger.io app collects active and passive data to support symptom monitoring for early psychosis patients. The study demonstrates an easy usability of the app and a willingness of patients to integrate apps into their care.
mEMA*^120,121^	Yes	Yes	−	+	−	Raugh et al. 2020^120^	The mEMA app conducts EMA surveys. The results indicate that passively collected geolocation in addition to the active data collected via the app are valid tools to measure negative symptoms and functional outcomes of schizophrenia patients.
mEMA(MACS)^122^	Yes	Yes	−	+	−	Moitra et al, 2021^122^	MACS, which runs through the mEMA platform, is a symptom and treatment adherence monitoring app. The study findings showed that the MACS intervention decreased dysfunctional coping strategies and symptoms.
MEmind*^123,124^	Yes	Yes	−	+	−	Berrouiguet et al. 2017^123^	The MEmind app serves as a clinical decision support system by analyzing clinicians’ antipsychotic prescription habits and monitoring changes in patients’ symptoms. The study results underscore the prevalence of antipsychotic prescription to inpatients with schizophrenia or related psychosis.
mindLAMP*^125–127^	Yes	No	−	+	−	Shvetz et al. 2021^125^	The mindLAMP app aims to measure cognitive impairments in schizophrenia patients using the Jewels Trail Test. The results showcase that the Jewels Trail Test provides an accessible and feasible tool to assess cognition of schizophrenia patients.
Monsenso(MindFrame)^128^	Yes	Yes	−	+	+	Terp et al. 2018^128^	MindFrame, which runs through the Monsenso platform, is a self-management app for young adults with schizophrenia that provide self-assessment, psychoeducation, action plan, medication management, and warning signs notifications. The study indicates that the app aids young adults to stay connected to symptom monitoring and medication adherence.
MovisensXS*^129,130^	Yes	No	−	+	−	Bell et al. 2018^129^	The MovisensXS app administers an EMA survey twice a day. The study aims to utilize the app in addition to TAU to enhance the coping strategy and reduce the severity of voice hearing experience in individuals with psychosis.
Proteus Biomedical*^131,132^	No	N/A	−	+	−	Reinertsen et al. 2017^131^	The Proteus Biomedical app receives heart rate and locomotor data from the sensor patch. The study results indicate that the passive heart rate and locomotor collection using the app may serve as a feasible tool to classify the severity of schizophrenia.
PsyMATE (ACT-DL)*^133–135^	Yes	Yes	+	+	−	Vaessen et al. 2019^133^	The ACT-DL app, built from the PsyMATE platform, aims to improve negative symptoms in schizophrenia patients by providing acceptance and commitment therapy in daily life. The study outcomes emphasize that participants found the app to be helpful in establishing skills and insights gained from their weekly therapy.
PsyMATE (SMARTapp)^136^	Yes	Yes	+	+	−	Hanssen et al. 2020^136^	The SMARTapp, built from the PsyMATE platform, provides ESM-derived tailored feedback to improve daily social functioning and symptoms of schizophrenia. The results indicate decreased momentary psychotic symptoms in SMARTapp group compared to TAU.
Purple Robot(SleepSight)*^137,138^	No	N/A	−	+	−	Meyer et al. 2018^137^	The SleepSight app, which runs through the Purple Robot platform, is used alongside a wearable Fitbit to track sleep data and collect sleep surveys. The results demonstrate high engagement with the wearable device and the app.
RealLife Exp*^139,140^	Yes	No	−	+	−	Kumar et al. 2018^140^	The RealLife Exp app administers daily mood and symptoms surveys for patients with early psychosis. The study outcomes emphasize the willingness of early psychosis patients to integrate the app into their current care.
Robin Z^141^	Yes	No	+	+	−	Traber-Walker et al. 2019^141^	The Robin Z app provides symptom management, medication tracker, crisis intervention plan, goal-setting, and positive reinforcement library to improve the functioning of individuals at CHR for psychosis. The study aims to improve daily functioning, self-efficacy, and quality of life following the Robin Z app intervention in addition to TAU.
SleepBot^142^	No	N/A	−	+	−	Berry et al. 2021^142^	The SleepBot app collects sleep movement and sound via accelerometer and microphone. The results show that the app is neither suitable nor valid to measure sleep activity in patients with schizophrenia.
Unnamed App^143^	No	N/A	−	+	−	Cinemre et al. 2022^143^	Cinemre et al conducted a pilot study for the use of an app, developed using the Apache Cordova platform, to track medication and activities adherence in schizophrenia patients. The results indicate that the overall use of the app decreased over time, and the app use reduced the PANNS and FROGS scores.
Unnamed App^144^	No	N/A	−	+	−	Parrish et al. 2021^144^	Parrish et al conducted a study for the use an app, developed using the NeuroUX platform, to provide ecological mobile cognitive tests and sessions designed for patients with serious mental illness, including schizophrenia. The study indicates that the app may be a suitable tool to assess cognitive functions.
Unnamed App^145^	No	N/A	−	+	−	Sigurðardóttir et al. 2022^145^	Sigurðardóttir et al conducted a study for the use of a smartwatch to collect accelerometer data, and a mobile app to administer wellbeing questionnaires to assist patients with bipolar disorder and schizophrenia. The study outcomes indicate that the patients were interested and willing to use the app and the smartwatch to monitor their data.
VS ePRO(BACS)*^146,147^	Yes	Yes	−	+	−	Atkins et al. 2017^146^	The BACS app, which is based on the VS ePRO app, is a tablet version of The Brief Assessment of Cognition in Schizophrenia. The study demonstrates that the BACS app results are consistent with that of the paper BACS.
WeChat*^148–150^	Yes	No	−	+	+	Zhu et al. 2020^148^	The WeChat app sends reminders for medication and psychoeducation messages to patients with schizophrenia. The study demonstrates increased medication adherence and improved quality of life.

*T* therapeutic, *M* monitoring, *P* psychoeducation, *mDOT* modified directly observed therapy, *mCBTn* mobile assisted cognitive behavioral therapy for negative symptoms, *TAU* treatment as usual, *MERIT* metacognitive reflection and insight therapy, *EMA* ecological momentary assessment, *ESM* experience sampling method, *LSN* letter-number span, *VPT* visual patterns test, *CHR* clinical high risk, *PANNS* positive and negative syndrome scale, *FROGS* functional remission of general schizophrenia scale, *CBT* cognitive behavioral therapy, ±: download unavailable in the US, *: articles with a maximum of two other representative articles.

### Research app accessibility

Regarding research app accessibility, 31 of the 60 research apps were not available on the public app marketplace. The remaining 29 research apps were available to be downloaded for free; however, 20 of the 29 research apps required an access code or special credential to access the app features. Thus, only nine research apps were available and easily accessible on the public app marketplace.

Research apps with psychosis-specific platform and non-specific platform differed significantly in their download availability on the public app marketplace (*χ*^2^ = 4.241, *p* = 0.039), with 64.5% of psychosis-specific platform apps and 37.9% of non-specific platform apps unavailable to be downloaded. Subsequently, research apps with non-specific platform had significantly higher download availability on the public app marketplace compared to that of research apps with psychosis-specific platform. However, both research apps with psychosis-specific platform and non-specific platform had limited accessibility with 29.0% and 37.9% of the apps requiring an access code, respectively (Table 4).

**Table 4:** Feature type and availability of psychosis research apps.

Variable	Platform type		
	Total *n* (%)	Specific *n* (%)	Nonspecific *n* (%)
*Total apps*			
	60 (100.0%)	31 (100.0%)	29 (100.0%)
*Feature type*			
T	7 (11.7%)	7 (22.6%)	0 (0.0%)
M	30 (50.0%)	8 (25.8%)	22 (75.9%)
P	0 (0.0%)	0 (0.0%)	0 (0.0%)
T and M	14 (23.3%)	10 (32.3%)	4 (13.8%)
T and P	3 (5.0%)	2 (6.5%)	1 (3.4%)
M and P	3 (5.0%)	1 (3.2%)	2 (6.9%)
T, M, and P	3 (5.0%)	3 (9.7%)	0 (0.0%)
*Availability*			
Not available	31 (51.7%)	20 (64.5%)	11 (37.9%)
Available with an access code	20 (33.3%)	9 (29.0%)	11 (37.9%)
Available without an access code	9 (15.0%)	2 (6.5%)	7 (24.1%)

*T* therapeutic, *M* monitoring, *P* psychoeducation.

## Discussion

Study results suggest that while many academic apps have been developed to support people with schizophrenia, very few suitable apps are actually available for use today. The situation is related to the commercial marketplaces where hundreds of apps are returned in searches but there are less than ten that are accessible and clinically relevant. Despite impressive research efforts, people with schizophrenia are today able to access a paucity of apps. A focus on translating research efforts into accessible apps should become the priority for the field.

We identified sixty unique apps used in schizophrenia research. Of these 60 apps, 31 (51.7%) were not available at all meaning that it is challenging to replicate or expand on their results. This result is perhaps not surprising given the challenges and costs associated with maintaining apps. For example, Krzystanek, a developer of MONEO app included in the results^31,32^, explains “the investor who wanted to commercialize it [the MONEO app] was not up to the task, so the project was suspended.”^33^ However, our results do suggest one potential solution as we found that apps developed on non-specific platforms were significantly more available today than those created on customized app platforms (*χ*^2^ = 4.241, *p* = 0.039). While a customized app offers clear advantages, using a broader platform may offer a more rapid and sustainable approach, especially for early phase work.

While there are many reasons a research app may not be available for easy public use, our result that 20 (33.3%) were also not accessible as they required an access code is notable given the goal of most apps is to increase access to care. While our results cannot directly support why, it is likely that these apps themselves are necessary to use in concert with a care program and not as standalone self-help tools. Using apps to augment care can certainly help increase access to care, but raises the need for concomitant training, workforce, implementation, and clinical infrastructure to support scalability. In reviewing the relevant literature, we found such documentation was often lacking although it represents an important target for new and ongoing app efforts.

Of the 60 research apps, only nine (15.0%) research apps were available to download and directly use. However, these results must be interpreted with caution as only two of these nine apps was created on a schizophrenia specific platform and the other seven on non-specific app platforms. Examples of non-specific platforms include WeChat, MovisensXS, and mindLAMP (developed by the authors) meaning that a patient or clinician would need to customize or add content to the platform for it to be ready for clinical use. This may often involve a licensing fee depending on the app or at least some degree of technical knowledge, reflecting a further barrier to access.

While there is room to improve accessibility of research apps, our results suggest this work is necessary as the current marketplace offerings are concerning. After an exhaustive search of apps on the Apple App Store and Google Play App Store, we found only 25 psychosis-specific marketplace apps from the 537 our search revealed. Even though there exist other curated third-party health app marketplaces (e.g., Psyberguide^34^) that provide information regarding app availability, there is no reason to believe that any missed marketplace apps would change our results. Of these 25 apps, only six were deemed clinically appropriate with the other 19 offering inaccurate, outdated, stigmatizing, or inappropriate content. For example, one app tells users to “remember that it’s [schizophrenia] all in your head.”^35^ Two apps recommend “St John’s Wort [which] works as an antidepressant in patients with schizophrenia disorder”^36,37^ as a part of the home remedies without clear warnings of dangerous medication interactions^38^. Three apps offered psychoeducation about “Disorganized, Catatonic and Undifferentiated schizophrenia”^39–41^ which have been removed from the DSM-5 for over five years^42^.

Considering the six (1.1% of 537 marketplace app) that were deemed clinically appropriate, five apps (only available on Google Play App Store) solely provided information/psychoeducation, and only one app (available on both Apple App Store and Google Play App Store) included therapeutic and monitoring features. The paucity of apps with therapeutic features may be that apps with therapeutic or diagnostic features are subject to Food and Drug Administration’s regulation as those apps do not fall under the “wellness” app category^43^. However, all six apps are not currently updated with an average time since last updated being 1121 days (3.07 years ago). Of note, while these apps were not updated recently, the psychoeducation they provided was so general that the content was not out of date. Still, considering that these are the apps most readily accessible to people with schizophrenia today it is clear that the potential of apps to increase access to care has yet to be fulfilled. Furthermore, the scarcity of clinically appropriate schizophrenia apps on Apple App Store compared to Google Play App Store raises a concern as this disparity can potentially induce health inequalities.

Our results suggest clear and tangible next steps. For research to accelerate to increase access to care, it is necessary to either build apps on non-specific platforms or to make apps built on specific platforms directly available for people to use. While advanced apps are being studied, there is a clear need to provide simple but up to date apps directly on the Apple and Android marketplaces so that people with schizophrenia can at least benefit from higher quality psychoeducation and app offerings.

Our results also suggest a broader question around when an app is necessary for schizophrenia specifically versus when a more transdiagnostic app will suffice. For example, medication tracking is a common feature across nearly every health condition and the added value of a schizophrenia specific vs general medication tracker remains an open question. Similarly, apps which help support people to engage in regular physical activity, adopt healthy eating patterns, quit smoking or even or manage common physical health conditions such as type-2 diabetes are theoretically just as important (or more so) for use in schizophrenia as the general population^44^. In our team’s work with the MIND website which evaluates apps, we often find ourselves helping people create toolkits of apps that utilize useful features across a range of apps to create the right set of resources for each patient. Research onto a common or transdiagnostic set of app functions for mental health, including schizophrenia, could help focus schizophrenia research on the unique challenges of the field and avoid unnecessary duplication of work.

Our findings expand on prior research results that support our results. Prior studies from our team have examined the top 10 apps for schizophrenia and found similar results^28,45^, but those studies did not exhaustively search for every app as we did here. Another review found that the number of commercially available apps with academic evidence from across all health fields is scarce but did not focus on schizophrenia and is based on older 2017 data^46^. Our results complement past reviews of research apps for people with schizophrenia^22,23^ that also found few available for patients although here we found nearly twice the number of research apps as compared to prior studies – perhaps reflecting accelerating interest in this space.

Like all reviews, ours has several limitations. We only search for research papers in English and are aware that given the many diverse names used to characterize this research, our search term may have missed some studies. However, a primary purpose of the review was to gain an assessment of the extent to which apps in readily-accessed research studies are aligned with those widely available on the marketplace, and there is no reason to believe that any missed studies more difficult to identify would reveal a preponderance of research apps in a way that would change our results. Our marketplace search was exhaustive, but we also realize searches from different regions (ie outside of the United States) may yield different results such as emerging work from China^47^. A search with more keywords may have included more apps but given only 1.1% of the returned results were relevant, we felt our search was already comprehensive.

## Data Availability

All data are presented in the paper or shared directly on mindApps.org
